# Intrauterine Hypoxia and Epigenetic Programming in Lung Development and Disease

**DOI:** 10.3390/biomedicines9080944

**Published:** 2021-08-02

**Authors:** Yajie Tong, Shuqing Zhang, Suzette Riddle, Lubo Zhang, Rui Song, Dongmei Yue

**Affiliations:** 1 Department of Pediatrics, Shengjing Hospital of China Medical University, Shenyang 110004, China; tongyj1@sj-hospital.org; 2 School of Pharmacy, China Medical University, Shenyang 110122, China; shawn22728@outlook.com; 3 Cardiovascular Pulmonary Research Laboratories, Departments of Pediatrics and Medicine, University of Colorado Anschutz Medical Campus, Aurora, CO 80045, USA; suzette.riddle@cuanschutz.edu; 4 Lawrence D. Longo, MD Center for Perinatal Biology, Department of Basic Sciences, Loma Linda University School of Medicine, Loma Linda, CA 92350, USA; lzhang@llu.edu

**Keywords:** hypoxia, lung development, epigenetics, developmental disorder, programming

## Abstract

Clinically, intrauterine hypoxia is the foremost cause of perinatal morbidity and developmental plasticity in the fetus and newborn infant. Under hypoxia, deviations occur in the lung cell epigenome. Epigenetic mechanisms (e.g., DNA methylation, histone modification, and miRNA expression) control phenotypic programming and are associated with physiological responses and the risk of developmental disorders, such as bronchopulmonary dysplasia. This developmental disorder is the most frequent chronic pulmonary complication in preterm labor. The pathogenesis of this disease involves many factors, including aberrant oxygen conditions and mechanical ventilation-mediated lung injury, infection/inflammation, and epigenetic/genetic risk factors. This review is focused on various aspects related to intrauterine hypoxia and epigenetic programming in lung development and disease, summarizes our current knowledge of hypoxia-induced epigenetic programming and discusses potential therapeutic interventions for lung disease.

## 1. Introduction

Intrauterine hypoxia, also known as fetal hypoxia, refers to a scenario where the fetus is deprived of an adequate oxygen supply due to environmental, maternal, placental, and fetal factors [1,2,3,4,5,6]. Excessive hypoxia can prevent the fetus from completing its genetically determined growth potential, leading to approximately 10% of babies stunted in the womb and delivered prematurely. The intrauterine hypoxia is involved in perinatal “plasticity,” affecting lung development, injury, and repair processes [6]. Bronchopulmonary dysplasia (BPD) is a multifactorial neonatal chronic lung disease, which is influenced by oxygen and/or intrauterine environment, genetics, immaturity, infection, poor nutrition, and mechanical ventilation. These risk factors are inter-related and interact during fetal development. For instance, prenatal hypoxia followed with postnatal hyperoxia rather than normoxia can exaggerate retardation of morphological lung development leading to BPD-like alterations [7,8]. In addition, excess chronic hypoxia followed by relative hyperoxic exposure, mechanical and infectious stimuli incite oxidative stress, contributing to BPD pathogenesis [9]. ROS can lead to cell apoptosis and dysfunction in alveolar cells, bronchial epithelial cells, alveolar macrophages, and endothelial cells by directly disrupting proteins, carbohydrates, lipids, DNA, and RNA [10]. Additionally, ROS can also trigger epigenetic modifications altering related gene expression patterns [11]. Both direct and epigenetic effects of ROS can cause impaired alveolar and capillary development, boosted vascular permeability and inflammatory responses, and promote the development of BPD [10,12].

This review focuses on the damage of excessive chronic intrauterine hypoxia to lung development and its related effects on the pathogenesis of BPD.

BPD is a chronic lung injury syndrome involving lung developmental plasticity, damage, and repair, which can limit the remodeling ability of surviving infants’ lungs until adulthood [13]. BPD is a breathing disorder in which the infants’ lungs become irritated and develop abnormally [14,15]. Although intensive treatment and support have improved the infants’ life prospects, morbidities related to severe BPD persist. The vulnerability of BPD has also evolved. “Old BPD,” defined in the 1960s, is characterized by severe lung injury. In premature infants showing reduced vascular and alveolar development with improved survival, “new BPD” was identified [16,17,18]. There is preclinical and clinical evidence that the new form of BPD is caused by early developmental arrest and impaired lung development rather than acute lung injury. This highlights the need for a better understanding of the molecular pathways that guide normal lung development and discover of the mechanisms and applications of lung regeneration [19,20,21,22].

Notably, oxygen-dependent epigenetic programming and regulation are the primary focus of intensive studies today. Epigenetic programming and modification cause inheritable and stable gene expression patterns without coding sequence alterations. Epigenetic mechanisms including DNA methylation, histone modifications, and miRNA expression play a significant role during hypoxia regulating different lineage-specific expression profiles and the transcriptional program for gene expression [5,6,23,24]. These are also the essential mechanisms for developmental programming. Similar studies have emphasized that epigenetic modifications and programming in response to environmental stimuli are critical in achieving appropriate gene expression patterns, especially in specific lung tissues in relation to environmental signals [25,26]. This review focuses on aspects associated with chronic intrauterine hypoxia and epigenetic programming in bronchopulmonary dysplasia. Taking into account the common growth and development disorders in BPD infants, any small growth amelioration may support pulmonary rehabilitation.

## 2. Epigenetics and Lung Development

**Figure 1:**
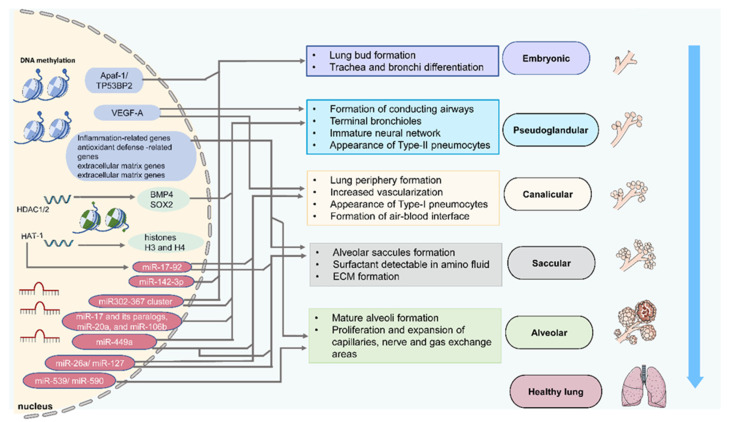
Epigenetic regulation of lung development. Schematic depicting of epigenetic components such as DNA methylation, histone acetylation, and miRNA expression to modify targeted genes and determine phenotypes at different lung development stages. These epigenetic components may have a crosstalk effect.

According to Yang and Schwartz [27], epigenetic mechanisms can control gene expression in chronic lung conditions such as idiopathic pulmonary fibrosis and chronic obstructive pulmonary disease. Although few studies have explored this hypothesis, the expectation is that research on epigenetics and lung development will become prominent in the near future. Epigenetics is considered the study of heritable changes in gene expression triggered by mechanisms that are separate from the expressed DNA sequence [28,29]. There are environmentally induced epigenetic alterations to DNA that can disrupt cells during development, leading to gene expression changes. Changes in the transcriptomes and epigenetics of lung cells occur at developmentally sensitive time points (Figure 1) (Table 1). Such changes in the epigenome of the cells may change the structure and function of the lungs. Although epigenome changes may result in subsequent changes in the epigenetic platform [30], leading to the deviation from normal lung development during lung repair and after injury, epigenetic alterations during lung development enable the epigenome to guide gene expression response to future damage and guide lung repair.

### 2.1. DNA Methylation

DNA methylation is a reversible DNA modification resulting from the addition of a methyl group to cytosines in nucleic acids [25,45,46]. DNA methylation is carried out by maintaining DNA methyltransferase (DNMT) 1 and the de novo methyltransferases DNMT3A and DNMT3B. Active DNA demethylation is initiated by ten-eleven translocation (TET) enzymes (TET1, TET2, and TET3) [47,48,49]. Fetal lungs begin to form four weeks after conception and continue to develop after birth. With the capability to develop into every cell type in the body, the pluripotent embryonic cells differentiate into specific cell lineages [46]. As cells develop into specialized types, they undergo epigenetic changes on histones and DNA, resulting in specific cell lineages. Therefore, DNA methylation is crucial and plays a vital role in cellular differentiation.

In human embryonic lung cells, the methylation of CpG islands was frequently found in the proximal promoter regions of TP53BP2 (tumor protein p53 binding protein 2) and Apaf-1 (apoptotic protease activating factor-1) [31]. Notably, the inhibition of methylation markedly upregulated Apaf-1 expression in human embryonic lung cells. Apaf-1 may play an essential role in DNA damage-induced apoptosis. Moreover, the CpG island-related proximal promoter regions of Apaf-1 and TP53BP2 are the transcription factor binding sites. Therefore, the methylation of Apaf-1 and TP53BP2 may inhibit embryonic morphogenesis and play a vital role in early lung development [31]. At the pseudoglandular/canalicular stage of lung development, CpG island methylation in the VEGF-A promoter of primary fetal distal lung epithelial cells was demonstrated to play a crucial role in the vascular growth of the cardiopulmonary system [35]. DNA methylation-regulated genes were verified during normal alveolar septation [43,50]. These genes are involved in inflammation (e.g., B-cell CLL/lymphoma 6, chitinase-like 3, chemokine [C-C motif] receptor 6, Ccl5, signal transducer and activator of transcription 4, CD209a antigen, histocompatibility 2, O region β locus, CD19 antigen, killer cell lectin-like receptor subfamily B member 1B, B-cell linker, SH2 domain-containing 1A, protein kinase C theta, and β-2 microglobulin), antioxidant defense (e.g., extracellular superoxide dismutase 3, and peroxiredoxin 6), extracellular matrix formation (Collagen alpha-3 (VI), Collagen alpha-1 (XXVII), elastin, tenascin C), and lung cancer (ribonucleotide reductases M 1, and 2, and S-phase kinase-associated protein 2) [43]. A more recent study identified numerous differentially methylated CpGs at birth that reflect fetal lung developmental processes [51]. These findings may contribute to our understanding of how the DNA methylation mechanism is related to normal and altered lung development.

### 2.2. Histone Acetylation

Histone modifications also play a critical role in the epigenetics of lung development and complements regulation by DNA methylation. Gene expression patterns can be controlled by a complex combination of histone acetylation and deacetylation [30,52]. For instance, Joss-Moore et al. [53] determined that the regulation of gene expression depends on the fine regulation of histone modifications. Rapid local changes in histone conformation take place during the acetylation of nucleosomes. While the mechanisms remain unelucidated, rapid deacetylation and acetylation could facilitate the transit of polymerases in nucleosomes [30]. Histone acetyltransferases (HATs) mediate the acetylation of histone tails, thereby promoting gene transcription, while histone deacetylases (HDACs) remove acetyl groups leading to gene silencing [54,55]. HAT1 is essential for the acetylation of newly synthesized histones H3 and H4. In Hat1^-/-^ mice embryonic fibroblasts are sensitive to DNA disruptors and exhibit high levels of genomic instability. In addition, HAT-1 deficiency can cause lung development defects, leading to neonatal mortality [32]. HDAC1/2 deficiency in proximal lung endoderm progenitors directly increased Bmp4 expression, subsequently decreased Sox2 expression, and inhibited the development of multiple proximal cells, contributing to defects in the proximal airways and lung morphogenesis [36].

### 2.3. MicroRNAs

MicroRNAs (miRNAs or miRs) are a class of small (18–25 nucleotides in length) noncoding RNAs. They usually inhibit gene expression by promoting mRNA degradation or disrupting its translation. Argonaute 1–4 and Dicer, two essential miRNA processing proteins, are expressed in the developing lung epithelium and mesoderm [56,57]. Notably, the loss of Dicer in lung epithelial cells can lead to severe defects in branching and epithelial structure, leading to perinatal death [58].

At the embryonic stage, miR-142-3p was found to be specifically upregulated in the embryonic lung mesenchyme. Furthermore, inhibition of miR-142-3p led to ectopic expression and differentiation of parabronchial smooth muscle cell progenitors in the mouse embryonic lung [33]. Similarly, miR-326 was found to play an essential role in the expansion of the distal epithelium and disruption of regular branching patterns and mesenchymal integrity in the embryonic lung [34].

At the pseudoglandular stage, miR-17 and its paralogs, miR-20a and miR-106b were highly expressed in the lung. The downregulation of miR-17, miR-20a, and miR-106b caused significant branching defects in embryonic lung epithelial explants [37]. The miR302-367 cluster was mainly expressed in the embryonic lung epithelium at the embryonic and pseudoglandular stages. This cluster promoted lung epithelial proliferation and suppressed subsequent differentiation through direct inhibition of the expression of tumor suppressors Rbl2 and Cdkn1a [38]. In addition, miR-449a was found to be upregulated at the pseudoglandular, canalicular and saccular stages, and decreased dramatically at birth. MiR-449a increased the Mycn and Sox9 mRNA levels, and the Ki-67 and SOX9 protein levels to stimulate distal epithelial progenitor proliferation and mucociliary differentiation, supporting an essential role of miR-449a in the mid-stages of lung development [39].

MiR-26a is highly expressed in the rat fetal lung, especially at the saccular stage [59]. The knockout of miR-26a-1/2 in the mouse promoted the formation of dilated lumens and aerated regions at the beginning of the canalicular stage, and maturation of the alveolar structure at the canalicular and saccular stages of lung development [41]. MiR-127 was also highly expressed in the late stage of fetal lung development and regulated terminal bud count and bud sizes in the developing lung [40]. MiR-17-92 can be downregulated by histone acetylation, promoting alveolar type 1 cell spreading and lung sacculation [42]. In the pseudoglandular and saccular stages of rat lung development, let-7a, miR-93, miR-125b-5p, miR-146b, miR-296, miR-1949, and miR-3560 were identified to be consistently expressed in the rat embryonic lungs; while miR-1949, miR-125b-5p, miR-296, and miR-93 were downregulated; and let-7a, miR-146b, and miR-3560 were upregulated [59]. A recent miRNA profiling of human and mouse postnatal alveolar tissue found fifty up- or downregulated miRNAs at the alveolar stage. Significantly, the inhibition of miR-539 and miR-590 markedly reduced alveolar development, with a reduction in radial alveolar count and lung compliance [44]. The above studies support the crucial role of miRNA in lung development and suggest the crosstalk among the epigenetic mechanisms, requiring further investigation.

Over time, epigenetics has become a critical aspect in determining lung development for fetuses and infants by considering the external environment and factors around the pregnant mother. From these studies on epigenetics and lung development of fetuses and infants growing up, some limitations or challenges arise, especially in understanding the role of epigenetic regulation in gene expression under different lung conditions.

## 3. Hypoxia and Vulnerability of Neonatal Chronic Lung Disease

Intrauterine hypoxia has significant impacts on fetal development, including breathing disorders. While high altitude, maternal smoking, chorioamnionitis, hypertension/preeclampsia, and intrapartum and post-placental complications all induce intrauterine hypoxia, there are limited reports on the production of bronchopulmonary dysplasia in such hypoxic conditions. In addition to being attributed to hypoxic injury, some of these risk factors (such as chorioamnionitis and preeclampsia) are complex pathological processes. Several clinical trials reported an association between higher altitude hypoxia and an increased prevalence of BPD [60,61]. Long-term hypoxia at high altitudes in pregnancy boosted ROS synthesis in ovine uterine arteries [62,63], providing additional evidence that hypoxia-induced ROS can perform a critical mechanistic part in the formation of irregular vasculature and BPD.

Unlike other organs, the lung completes a fraction of its development immediately before and after birth. During the final lung development stage (alveolarization), the secondary septation process breaks down alveolar ducts to alveolar sacs, including the expansion of capillary beds through angiogenesis to increase the lung’s surface area to facilitate gas exchange [17,64]. However, the growth completion in the postnatal stage means that the lung becomes highly vulnerable to external factors or environmental pressures, which interfere with the developmental program [53], this is particularly evident in the preterm birth setting, where alveolarization disruption results in BPD, which is commonly known to be the most common maturity complication [17,65,66]. The evolution of the lung’s vascular system resembles that of the airways, including peribronchial arteries rising and separating concurrently with the airways. Once splitting is done, angiogenesis and vasculogenesis join to create the alveolar-capillary bed inside the fetal lung mesenchyme. The canalicular and saccular levels of lung growth are when the alveolar capillary bed is formed [67]. Throughout capillary creation, epithelial growth factors such as VEGF, which is regulated by hypoxia/HIF-1α and other signaling, recruit forming capillaries towards the epithelial basement membrane, facilitating epithelial–endothelial interactions and the creation of alveolar septa in the late alveolar developmental phase [68]. Latest research indicates that interruption of normal lung vascular development can be a critical factor in the pathogenesis of BPD [69,70,71]. While lung vascular formation has been studied extensively in alveolar stage animal models, all hypoxia and hyperoxia result in emphysema with decreased alveolar numbers and capillary density in the alveolar stage [72,73]. It is unknown if these models accurately replicate BPD pathogenesis because newborns during the late saccular and alveolar developmental stages frequently experience BPD. The essential mechanisms underlying BPD, including possible vasculature dysregulation in the developing lung, most likely begin during the canalicular and saccular phases of lung growth. Thus, it is assumed that persistent hypoxia has an adverse effect on the pulmonary blood vessels, leading to BPD, which mainly occurs in the tubule/sac phase and may also be at the alveolar level. As is often the case, infants battling BPD require respiratory support in the early stages of infant life failure. Mechanical ventilation and high concentration oxygen supply can cause hyperoxia injury, which enhances hypoxia-induced lung developmental disorder. A considerable population would experience long-term deficits in pulmonary function, including persistent airway obstruction and delayed growth of the distal lungs.

**Figure 2:**
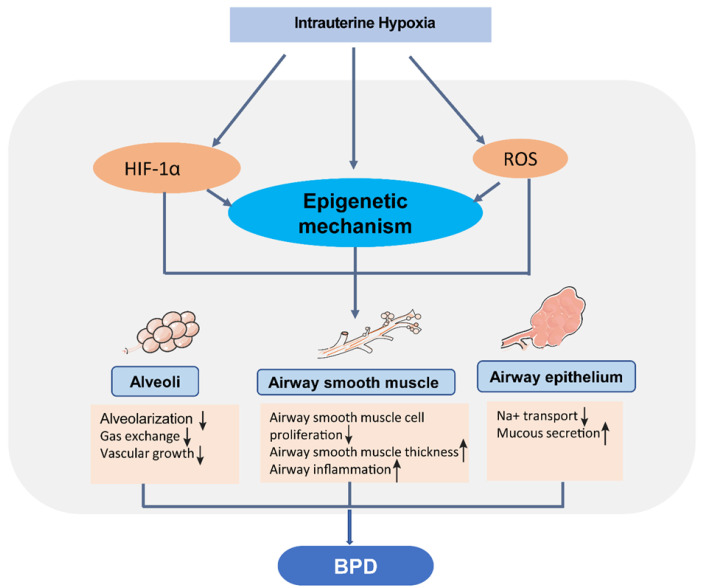
Intrauterine hypoxia increases the susceptibility to BPD. Intrauterine hypoxia results in different pathophysiological phenotypes of the alveoli (for example, defects in alveolarization, gas exchange, and blood vessel growth), airway smooth muscle (for example, reduction of airway smooth muscle cells proliferation and increased airway smooth muscle thickness and inflammation) and airway epithelium (for example, decreased Na^+^ transport and increased mucus secretion). These structural or functional defects mediated by hypoxia signals lead to infant BPD susceptibility.

To further understand the vulnerability to BPD, it is crucial to determine how the lung develops. As mentioned above, there are various stages of lung development. The alveolar stage begins before birth and extends up to some years after birth [17,64,74]. Chronic hypoxia impairs alveolarization and lung development [7,75,76]. Pulmonary angiogenesis and secondary septation markedly increase the lung’s surface area for gas exchange [17,27,64,77,78]. The early lung development stages involving the development of pulmonary vasculature appear to take place mainly through vasculogenesis [64,67]. Under intermittent hypoxia (12% O_2_), angiogenic gene and protein expression were downregulated, and thus, angiogenesis and lung development were perturbed in newborn mice [79,80]. At the same time, the secondary septation process is an integrated, complex series of events that involve paracrine signals between different cell types within the lung, and comprises epithelial cells, fibroblasts, endothelial cells, and inflammatory cells [81,82,83]. Taken together, these stages and the possible impact of the disease on the lungs illustrate their vulnerability under intrauterine hypoxia (Figure 2).

## 4. Epigenetic Programming and Neonatal Chronic Lung Disease

Epigenetics influences cell-specific developmental gene transcription and gene silencing. There is a timely requirement for gene transcription regulation during normal development. In this case, only the genes that are specific to a particular cell type and developmental stage are transcriptionally active [84,85]. The ability to conduct gene transcriptional modulation provides “plasticity” during development [86,87]. Dysregulation of chromatin remodeling pathways, which include DNA methylation, miRNA regulation, and histone acetylation, has also been identified in recent studies as part of epigenetic programming in response to hypoxia or hypoxia/hyperoxia in neonatal or infant lungs, different experimental models, and BPD patients [88,89,90,91,92,93] (Figure 2) (Table 2).

To determine the impact of epigenetic programming on lung development and BPD, epigenetic modifications assist in directing the associated factors and transcriptional machinery to the correct location within genes. One of the primary and better understood epigenetic programming features is DNA methylation. Here, the emphasis is on the alveolar septation process in neonatal lungs, accompanied by changed DNA methylation profiles coinciding with distinct gene expression changes. DNA methylation profiling identified 149 differentially methylated genes in lung tissue samples from preterm infants with BPD [43]. Out of 149 genes with altered methylation status, 23 genes had opposite patterns of methylation and expression. Among them, the methylation of zinc-finger protein 438 was decreased and associated with an increased expression in BPD, while the other genes displayed increased methylation corresponding with decreased expression in BPD [43]. Further analysis demonstrated that pathways associated with genes differentially methylated and expressed in BPD included ErbB signaling, neuregulin signaling, RhoA signaling, VEGF signaling, cardiomyocyte differentiation via BMP receptors, axonal guidance signaling, and glutathione-mediated detoxification [43]. Some of those pathways such as RhoA signaling, axonal guidance signaling and glutathione-mediated detoxification have been implicated in lung development and BPD pathogenesis [94,95,96].

**Table 2:** MiRNAs in hypoxia and BPD.

miRNA	Regulation	Species and Samples	Targets	Disease/Condition	References
miR-17-92	Downregulated	Human infant lungs	?	Extremely and very preterm, BPD	[97]
miR-103a-3p and miR-185-5p miR-200a-3p	Downregulated Upregulated	Umbilical cord blood-derived exosomes from human infants	PI3K/Akt and angiogenesis-related signaling pathways	Very preterm, BPD	[98]
miR-15a	Upregulated	Chicken lung	Bcl2	Hypoxia	[99]
miR-210 and miR-374a	Upregulated	Plasma of newborn piglets	?	Hypoxia	[100]
miR-34a	Downregulated	Mouse lungs	?	Postnatal hypoxia-induced BPD	[101]

Other than DNA methylation, histone marker modifications, especially on the outer promoter regions, are important for the epigenetic programming of gene expression [102,103,104] and play a crucial role in the developmental origin of lung diseases [105,106,107,108,109]. DNA methylation of the promoter of miR-17-92 downregulated this miRNA cluster expression and mediated the molecular pathogenesis of bronchopulmonary dysplasia [97]. Indeed, miRNAs are also essential in mediating developmental lung disorders [110,111,112,113,114,115]. In the chicken lung, hypoxia stress stimulates miR-15a expression to direct target Bcl2 and inhibit its antiapoptotic activity at particular stages, which may be a novel therapeutic target for developmental lung disorders by hypoxia insults [99]. Saugstad et al. demonstrated that global hypoxia-ischemia in newborn piglets increased circulating miR-210 and miR-374a expression [100]. These two miRNAs were involved in the pulmonary vasculature and pulmonary hypertension and intrauterine growth restriction [116,117], which are closely associated with BPD. A more recent study found that umbilical cord blood-derived exosomal miRNAs from very preterm human infants with BPD were 90 downregulated (e.g., miR-103a-3p and miR-185-5p) and 328 upregulated (e.g., miR-200a-3p), which were associated with PI3K/Akt and angiogenesis-related signaling pathways [98]. Further, overexpression of miR-103a-3p and miR-185-5p augmented endothelial cell proliferation, migration and tube formation, while overexpression of miR-200a-3p impeded these angiogenic responses [98]. Additionally, miR-34a expression was found to be decreased in the postnatal hypoxia-induced mouse model of BPD/lung injury [101]. Although miR-34a was demonstrated to be upregulated in hyperoxia-induced mice BPD model and human BPD samples [101], it remains unclear the role of miR-34a and the downstream molecular mechanism in hypoxia-induced BPD. In addition, human BPD may be caused by both prenatal hypoxia and postnatal hyperoxia, so it deserves further investigation of the mechanistic role of miR-34a in prenatal hypoxia-induced BPD.

Recent studies in certain animal models, including intrauterine hypoxia, unveiled significant changes in gene expression patterns in the placenta and fetal organs/tissues (such as the heart, brain, cerebral artery, liver, pulmonary artery, and lung). It has been demonstrated that both HIF-1α and hypoxia-derived reactive oxygen species (ROS) can significantly mediate hypoxia-induced epigenetic programming and developmental disorders [6,118,119,120,121,122]. Although hypoxia-modulated epigenetics have been investigated in the placenta, fetal brain, and fetal heart, it remains unclear in the fetal lung. Further research is needed to investigate the effect of intrauterine hypoxia on global DNA and gene-specific DNA methylation patterns and other epigenetic patterns in the developing lung and understand how fetal stressors affect epigenetic changes at specific sites of associated genes in intrauterine hypoxia-related developmental diseases.

## 5. Perspectives for Treatment and Prevention of Neonatal Chronic Lung Disease

Despite the high prevalence of BPD, no effective therapeutics were available to treat this disease in the past twenty years. Only vitamin A, caffeine, and corticosteroid are clinically used after birth for BPD intervention [123,124,125,126]. Recently, research on the epigenetic involvement in BPD has been steadily increasing. As discussed above, epigenetic modifications, such as DNA methylation, histone acetylation, and miRNAs alteration, play important roles in BPD. Notably, some studies revealed that DNMT and HDAC inhibitors, or correcting miRNA expression could improve pathophysiological dysfunction in BPD [127,128,129,130]. Despite recent advances in the epigenetic field, clinical epigenetic therapy for BPD is still a challenge for future research. The mechanistic role of epigenetics in the occurrence and development of BPD is not completely understood, especially in pathological processes provoked by intrauterine hypoxia in the different stages of lung development.

A preliminary human miRNA profiling study in blood from preterm infants identified that miR-133b and miR-7 were more highly expressed in subjects with BPD compared to those without BPD, whereas the expression of miR-152 and miR-30a-3p decreased in the subjects with BPD. Furthermore, the downregulation of miR-152 and miR-30a-3p and upregulation of miR-133b and miR-7 were found in the BPD group, compared to the non-BPD group in the older-age set [93]. Therefore, more studies examining hypoxia-related epigenetic pathway alterations in the development of BPD the lung tissue and blood of different age sets will be necessary. This could provide evidence regarding the mechanical roles of the epigenetic modifications in the development of BPD and contribute to developing epigenetic pharmacological therapies.

**Figure 3:**
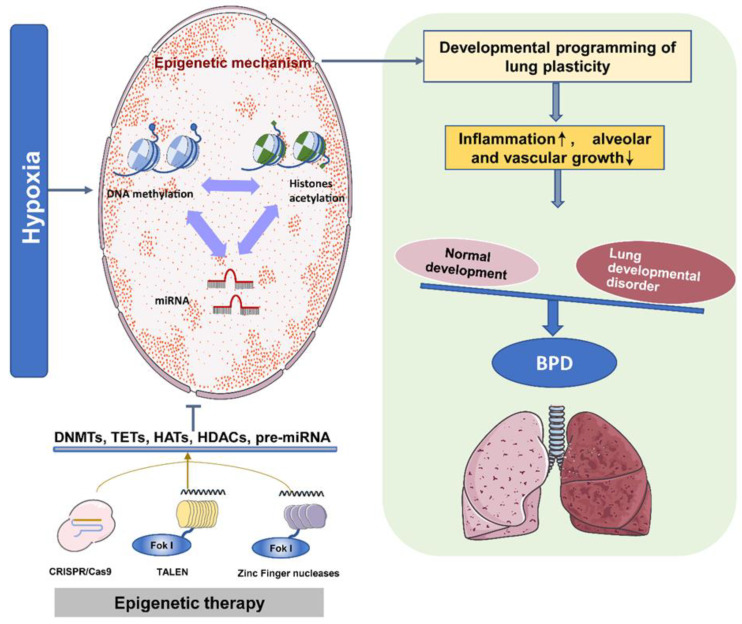
The epigenetic program controls the development of BPD. Under intrauterine hypoxia, different epigenetic mechanisms coordinate the development of lung plasticity. Therefore, inflammation is induced during lung development, and the growth of alveoli and blood vessels is inhibited. Eventually, normal and abnormal lung development are unbalanced, leading to the development of BPD. Epigenetic therapy based on DNMT inhibitors, HDAC inhibitors, and miR modulators can improve lung development and reduce the pathogenesis of neonatal chronic lung disease.

In the past 20 years, clinical trials have made significant progress in prenatal gene therapy and have provided hope for patients who suffered from genetic diseases. These diseases occur before or shortly after birth and may cause severe morbidity or death in the uterus or after birth. Moreover, there is no specific and efficient postpartum treatment method. Because gene transduction is easy to attain in fluid-filled fetal lungs, they are ideal for prenatal gene therapy [131]. A recent study demonstrated that the knockout of HDAC3 improved alveolarization and pulmonary angiogenesis in BPD [130]. The knowledge gained from studies in epigenetic modification-based postnatal gene therapy is insufficient, but it is a necessary starting point and a potential and promising strategy for treating and preventing BPD. In addition, evolving gene editing approaches, including the use of nucleases such as TALENs (transcription activator-like effector nucleases), zinc-finger nucleases, and CRISPR-Cas9 (clustered regularly interspaced short palindromic repeats-CRISPR-associated 9) to instigate a sequence-specific change in the DNA [132,133,134], encouraged the development of gene expression regulation, epigenetic modification, functional gene screening, gene diagnosis, and therapeutic drug discovery [135,136,137,138,139,140]. For example, dCas9 can be fused with DNMTs or TETs to regulate DNA methylation, thereby regulating specific gene expression [141,142,143]. Similarly, dCas9 can be fused with HDACs or HATs to regulate chromatin structure, thereby regulating gene expression [144,145,146]. Moreover, miRNA expression can be regulated by targeting the terminal loop or 5′ region of pre-miRNA by CRISPR-Cas9 technology [147,148,149]. The utero gene editing has been investigated in lung diseases [150,151,152,153]. Therefore, recent proof-of-concept studies support the feasibility of prenatal gene editing with high specificity for the lung and provide evidence that prenatal gene editing is potent and promising for the management of the developmental origin of lung diseases (Figure 3).

Nutritional supplement therapy has also been developed as a potential intervention for BPD, and various randomized clinical trials conducted in recent years have supported this approach. It is fascinating that many biologically active dietary components (“epigenetics diet”) change the epigenome [154,155]. These epigenetics diets include polyphenols (epigallocatechin-3-gallate, resveratrol, genistein), vitamins (vitamins c and d, folate, choline), isothiocyanates, withaferin a, and selenium. Perinatal vitamin D deficiency and low vitamin D levels have played a crucial role in neonatal BPD [156,157]. Significantly, vitamin D can directly stimulate fetal pulmonary artery endothelial cell and alveolar epithelial type II cell growth and function to prevent BPD development in preterm infants [158]. Dose- and time-dependent, and sex- and genetic background-specific epigenetic diets and their epigenome modification roles should be further investigated in BPD development.

Because the pathogenesis of alveolarization, vascularization, and tissue repair in immature lungs that cause BPD is multifactorial, the combined treatment of epigenetics and specific gene targeting methods will provide a broader prospect for the prevention and treatment of BPD.

Rodent-based forms of BPD provide significant advances in respect of genetic tools accessibility. Despite this, prenatal hypoxia, especially in preterm infants, and hyperoxia risk factors are far less significant clinical frameworks in animal BPD models. In addition, There there are limited utility for examining breathing dynamics, gas exchange, and pulmonary hemodynamics [159]. There seems to be an urgent need to adapt current models to more accurately reflect the pathological mechanisms at work in affected fetus and newborn infant, and conduct more rigorous evaluations of possible eligible pharmacological and nonpharmacological therapies for the treatment of BPD.

## 6. Concluding Remarks

Epigenetics plays a significant role in lung development. Lung development and the response to a conditioning injury (i.e., intrauterine hypoxia) require coordinated and structured proliferation and migration. For instance, lung development includes phenotypic modulation and programming to develop cells with specialized functions.

A better understanding of environmental influences (e.g., intrauterine hypoxia) and “developmental plasticity” on neonatal and fetal lung development will provide insights to establish preventive strategies to reduce or prevent abnormal lung development and the developmental origins of lung diseases.

## Figures and Tables

**Table 1:** Lung development and epigenetic regulatory components.

Stage	Species and Samples	Epigenetics	Targeted Genes	Characteristic Events	References
Embryonic	Human embryonic lung cells	DNA Methylation	TP53BP2 and Apaf-1	Cell proliferation	[31]
	Mouse embryonic fibroblasts	HAT-1	Histones H3 and H4	Embryonic lung development	[32]
	Mouse lung primordia	miR-142-3p	WNT signaling	Lung mesenchymal cells proliferation and differentiation	[33]
	Mouse embryonic lung explants	miR-326	Smo and Gli2	Lung mesenchymal cells proliferation and differentiation	[34]
Pseudoglandular	Rat fetal distal lung epithelial cells	DNA Methylation	VEGF-A	Airway and vascular branching	[35]
	Mouse proximal lung endoderm progenitors	HDAC1/2	BMP4/SOX2	Branching morphogenesis	[36]
	Mouse embryonic lung epithelial explants	miR-17 and its paralogs, miR-20a, and miR-106b	Stat3 and Mapk14	Epithelial bud morphogenesis	[37]
	Early lung endoderm	miR302–367 cluster	Rbl2 and Cdkn1a	Lung epithelial proliferation	[38]
	Human, murine, and avian fetal lungs	miR-449a	Mycn and Sox9	Epithelial proliferation and mucociliary differentiation	[39]
	Rat fetal lungs	miR-127		Lung branching	[40]
Canalicular	Rat fetal distal lung epithelial cells	DNA Methylation	VEGF-A	Airway and vascular branching	[35]
	Human, murine, and avian fetal lungs	miR-449a	Mycn and Sox9	Epithelial proliferation and mucociliary differentiation	[39]
	Mouse fetal lungs	miR-26a	SFTPA1, SFTPB, SFTPC	Formation of dilated lumens and aerated regions, maturation of the alveolar structure	[41]
Saccular	Human, murine, and avian fetal lungs	miR-449a	Mycn and Sox9	Epithelial proliferation and mucociliary differentiation	[39]
		miR-26a	SFTPA1, SFTPB, SFTPC	Maturation of the alveolar structure	[41]
	Mouse fetal lungs	HDAC3/miR-17-92	TGF-β	Alveolar type 1 cell spreading and lung sacculation	[42]
Alveolar	Mouse newborn lung	DNA Methylation	?	Alveolar septation	[43]
	Mouse postnatal and early child lung	miR-539 and miR-590		Alveolar development	[44]

## Data Availability

Not applicable.
